# Tropospheric NO_2_ and O_3_ Response to COVID‐19 Lockdown Restrictions at the National and Urban Scales in Germany

**DOI:** 10.1029/2021JD035440

**Published:** 2021-09-27

**Authors:** Vigneshkumar Balamurugan, Jia Chen, Zhen Qu, Xiao Bi, Johannes Gensheimer, Ankit Shekhar, Shrutilipi Bhattacharjee, Frank N. Keutsch

**Affiliations:** ^1^ Environmental Sensing and Modeling Technical University of Munich (TUM) Munich Germany; ^2^ School of Engineering and Applied Science Harvard University Cambridge MA USA; ^3^ Department of Environmental Systems Science ETH Zurich Zurich Switzerland; ^4^ Department of Information Technology National Institute of Technology Karnataka Surathkal India; ^5^ Department of Chemistry and Chemical Biology Harvard University Cambridge MA USA

**Keywords:** COVID‐19, emission reduction, GEOS‐Chem, nitrogen oxide, ozone, NO_X_‐saturated

## Abstract

This study estimates the influence of anthropogenic emission reductions on nitrogen dioxide (${NO}_{2}$) and ozone ($O_{3}$) concentration changes in Germany during the COVID‐19 pandemic period using in‐situ surface and Sentinel‐5 Precursor TROPOspheric Monitoring Instrument (TROPOMI) satellite column measurements and GEOS‐Chem model simulations. We show that reductions in anthropogenic emissions in eight German metropolitan areas reduced mean in‐situ (& column) ${NO}_{2}$ concentrations by 23 % (& 16 %) between March 21 and June 30, 2020 after accounting for meteorology, whereas the corresponding mean in‐situ $O_{3}$ concentration increased by 4 % between March 21 and May 31, 2020, and decreased by 3% in June 2020, compared to 2019. In the winter and spring, the degree of ${NO}_{X}$ saturation of ozone production is stronger than in the summer. This implies that future reductions in ${NO}_{X}$ emissions in these metropolitan areas are likely to increase ozone pollution during winter and spring if appropriate mitigation measures are not implemented. TROPOMI ${NO}_{2}$ concentrations decreased nationwide during the stricter lockdown period after accounting for meteorology with the exception of North‐West Germany which can be attributed to enhanced ${NO}_{X}$ emissions from agricultural soils.

## Key Points

- During the COVID‐19 lockdown period, NO_2_ concentrations decreased and O_3_ concentrations increased in eight German cities
- The degree of NO_X_ saturation of ozone production is weakening from winter to summer
- Meteorological variability adjusted by GEOS‐Chem model simulations driven by the same emissions for 2020 and 2019

## 1 Introduction

The outbreak of the novel COVID‐19 virus in late 2019 prompted governments to take various measures to prevent the COVID‐19 virus from spreading through society. These actions include physical distancing, a ban on large group gatherings, home office work, and international and domestic travel restrictions (DW COVID‐19, 2020). These measures resulted in a significant reduction in emissions following economic activity and overall mobility (Evangeliou et al., 2021; Gensheimer et al., 2021; Guevara et al., 2021; Le Quéré et al., 2020; Z. Liu, Ciais, et al., 2020; Z. Liu, Deng, et al., 2020; Turner et al., 2020). There has been a lot of interest in studying this time window and its impacts on the Earth system. Numerous studies (Bauwens et al., 2020; Berman & Ebisu, 2020; Chauhan & Singh, 2020; Collivignarelli et al., 2020; Dietrich et al., 2021; He et al., 2020; Keller et al., 2021; R. Zhang et al., 2020) have reported a reduction in major air pollutant concentrations during the COVID‐19 lockdown period, including nitrogen dioxide (${NO}_{2}$), carbon monoxide (CO), sulfur dioxide (${SO}_{2}$) and particulate matter (${PM}_{10}$ and ${PM}_{2.5}$), which are primarily emitted by fossil fuel consumption. During the COVID‐19 lockdown period, air quality improved in most countries, particularly in urban areas (Bedi et al., 2020; Fu et al., 2020). Previous studies, such as Bauwens et al. (2020); Deroubaix et al. (2021), compared lockdown period concentration with long‐term mean to estimate lockdown effects by eliminating the average climatological seasonal cycle. However, a direct comparison of lockdown period pollutant concentrations with pre‐lockdown period pollutant concentrations includes both meteorological and COVID‐19 emission reduction influences.

Meteorological effects must be considered to determine the actual impact of anthropogenic emission reductions on changes in pollutant concentrations during the COVID‐19 lockdown period (Barré et al., 2020; Deroubaix et al., 2021; Gaubert et al., 2021; Goldberg et al., 2020; Petetin et al., 2020; Sharma et al., 2020; Y. Liu et al., 2020), particularly with regard to chemical processes (Kroll et al., 2020). An analysis of pollutant concentration changes over the European networks of surface air quality measurement stations was performed to isolate the lockdown impacts based on a data‐driven meteorological adjustment (Ordóñez et al., 2020; Venter et al., 2020). Previous works (Gaubert et al., 2021; Menut et al., 2020; Mertens et al., 2021; Potts et al., 2021; Weber et al., 2020) have used different modeling approaches to investigate the impact of lockdown measures on air quality over Europe. The 2020 emission reduction scenarios were set up using available activity data from various sources (Doumbia et al., 2021; Forster et al., 2020; Guevara et al., 2021). As part of its modeling work, Gaubert et al. (2021) compared the 2020 lockdown period with climatological mean in order to separate the anomalies caused by the weather conditions in 2020, and they have called for more meteorology adjusted studies to avoid the flawed results.

We focus on nitrogen dioxide (${NO}_{2}$) and ozone ($O_{3}$) concentration changes due to 2020 COVID‐19 lockdown restrictions, from March 21 to June 30. We consider ${NO}_{2}$ and $O_{3}$ together from the perspective of atmospheric chemistry, because ${NO}_{2}$ and $O_{3}$ concentrations are functions of each other (Bozem et al., 2017). Nitrogen oxide (${NO}_{X}$ = NO+${NO}_{2}$) emissions have a pronounced seasonal cycle, with higher emissions in the winter than in the summer (Beirle et al., 2019; Kuenen et al., 2014). Half of the ${NO}_{X}$ in the troposphere is from fossil fuel consumption in urban areas (e.g., Figure S1). Tropospheric ${NO}_{2}$ concentrations follow a similar annual cycle, with higher values in the winter than in the summer. This is due to the fact that in addition to the higher emissions mentioned above also the lifetime of ${NO}_{2}$ is longer in the winter (≈21 h) than in the summer (≈6 h) (Shah et al., 2020). Peak ${NO}_{2}$ concentrations in the winter are also influenced by atmospheric inversion conditions. ${NO}_{2}$ influences climate by acting as a precursor to the formation of tropospheric $O_{3}$ (Crutzen, 1988; Jacob, 1999), and both ${NO}_{2}$ and $O_{3}$ have an impact on human health. Tropospheric ozone production is complex and depends strongly and non‐linearly on nitrogen oxides (${NO}_{X}$) and volatile organic compound (VOC) concentrations, despite the fact that photolysis of ${NO}_{2}$ is the only chemical source of tropospheric ozone (Council et al., 1992; Kleinman, 2005; Lin et al., 1988). Ozone decreases as ${NO}_{X}$ decreases in regions with low ${NO}_{X}$ and high VOC concentrations, that is, ${NO}_{X}$ limited regimes; however, in high ${NO}_{X}$ regions, that is, VOC limited regimes (or ${NO}_{X}$ saturated regimes), a decrease in ${NO}_{X}$ results in an increase in $O_{3}$ concentration (Kleinman et al., 1997; Sillman, 1999; Sillman et al., 1990) (Figure S2).

This study uses the TROPOspheric Monitoring Instrument (TROPOMI) on the Sentinel‐5 Precursor (Sentinel‐5P) satellite and governmental in‐situ ${NO}_{2}$ measurements as a proxy for changes in ${NO}_{2}$, and governmental in‐situ $O_{3}$ measurements as a proxy for changes in $O_{3}$ concentrations in Germany. To account for the impact of meteorology, we use the same anthropogenic emissions in 2020 and 2019 with 2019 open fire emissions and long‐term (1995–2013) monthly lightning ${NO}_{X}$ emission climatology for the GEOS‐Chem model. We are therefore able to present separate quantitative results for changes in ${NO}_{2}$ and $O_{3}$ concentrations caused by meteorological changes and by reductions in anthropogenic emissions resulting from COVID‐19 lockdown measures. To the best of our knowledge, no such study using GEOS‐Chem (GC) modeling to account for meteorological impacts has been conducted for Germany.

## 2 Study Regions, Data Sets, Model and Method

Our study region covers a bounding box over the national area of Germany (5–15.5°E, 47–55.5°N), with a particular focus on eight urban areas spread across the country: Munich, Berlin, Cologne, Dresden, Frankfurt, Hamburg, Hanover, and Stuttgart (Figure S3). This study mainly focused on the urban scale to examine the impact of reduced mobility on ${NO}_{2}$ and $O_{3}$ concentrations during the 2020 COVID‐19 pandemic period. We also extended our study nationwide to investigate other significant ${NO}_{X}$ sources in rural locations.

We used tropospheric ${NO}_{2}$ column data from the TROPOMI aboard the Sentinel‐5P satellite (Copernicus, 2020). The satellite is in a sun synchronous orbit with an equatorial crossing time of 13:30 (local solar time). TROPOMI ${NO}_{2}$ data has a spatial resolution of 7 × 3.5 km (increased to 5.5 × 3.5 km after August 6, 2019) and it covers the globe daily due to its wide swath (Van Geffen et al., 2020). TROPOMI ${NO}_{2}$ precision (error estimate originating from the spectral fit and other retrieval aspects) for each pixel is within the range of 3.6 × 10^14^ to 3.7 × 10^16^ molec. cm^−2^ (about 21%–75% of tropospheric ${NO}_{2}$ column). The TROPOMI ${NO}_{2}$ measurements for winter are highly uncertain (Figure S4). The main source of uncertainty is the calculation of the air mass factor, which is estimated to be on the order of 30% (Lorente et al., 2017). Since our study is mainly focusing on the relative difference in ${NO}_{2}$ between 2020 and 2019, the systematic errors associated with TROPOMI retrievals (e.g., spectroscopic errors and instrument bias) should cancel out, while random error component is persistent. However, when we apply temporal and spatial averaging, random errors are reduced. We followed S5P NO2 Readme (2020) for the quality filter criteria, which removes cloud‐covered scenes in order to avoid high error propagation through retrievals. We averaged the TROPOMI values within a radius of 0.5° from the urban center to create time series (& daily observations) at the urban scale. For comparisons between 2020 and 2019 at the national scale, TROPOMI tropospheric ${NO}_{2}$ column densities were gridded in 0.25 × 0.25‐degree bins.

We investigate agricultural activities in Germany using ammonia (${NH}_{3}$) data (Kuttippurath et al., 2020). The “Standard monthly IASI/Metop‐B ammonia (${NH}_{3}$) data set” was downloaded from IASI NH3 (2020). This data set contains monthly averaged ${NH}_{3}$ measurements (total column), from the Infrared Atmospheric Sounding Interferometer (IASI), onboard the Metop satellites, at 1 × 1° resolution. We also used the “Near‐real time daily IASI/Metop‐B ammonia (NH3) total column data set (ANNI‐NH3‐v3)” product to investigate the inter‐annual short‐term (less than a month) variability in ${NH}_{3}$ over Germany (IASI NH3, 2020).

In‐situ surface ${NO}_{2}$ and $O_{3}$ concentrations were obtained as hourly averaged measurements from the UBA's (German Environment Agency) database (Umweltbundesamt, 2020). We collected data from 38 stations in eight German cities, including both urban and rural measurement sites, for 2020 and 2019. In this study, we averaged all 24‐h measurements from stations located within each city.

The ERA5 data set (Copernicus Climate Change Service (C3S), 2017) is used as a reference data set to discuss meteorological conditions over study areas. We used the “ERA5 hourly data on pressure levels” product for wind speed and direction and temperature. Further, we used the “ERA5 hourly data on single levels” product for boundary layer height. We averaged these values within a radius of 0.5° from the urban center to create a time‐series (& daily observations) at the urban scale. The sunshine duration (hours per day) data was obtained from Deutscher Wetterdienst (DWD, 2020).

The GC chemical transport model (GEOS‐Chem, 2020) is used to estimate the concentration differences in ${NO}_{2}$ and $O_{3}$ between 2020 and 2019 caused by meteorological changes. The GC model is driven by MERRA‐2 assimilated meteorological data (MERRA‐2, 2020). We conduct nested simulations over Germany (4‐17°E, 45‐57°N) at a horizontal resolution of 0.5° × 0.625° with dynamic boundary conditions generated from a global simulation by 4° × 5° resolution. GC assumes the same anthropogenic emissions in 2020 and 2019. We used anthropogenic emissions in 2014 from the Community Emissions Data System (CEDS) inventory (Hoesly et al., 2018) and 2019 open fire emissions from GFED4 (Werf et al., 2017) for both 2019 and 2020 simulations. The spatial and monthly climatology of lightning ${NO}_{X}$ emissions is constrained by LIS/OTD satellite observations averaged over 1995–2013. We used an improved parameterization approach implemented in the GC model to calculate soil ${NO}_{X}$ emissions (Hudman et al., 2012). In all comparisons of the GC model to TROPOMI, GC ${NO}_{2}$ vertical profile simulations (at 47 vertical layer) are converted to ${NO}_{2}$ column densities for TROPOMI footprints by interpolating into TROPOMI measurements pressure levels and applying TROPOMI's averaging kernels. Similar to above, GC column densities were gridded in 0.25 × 0.25‐degree bins at the national scale.

Our methodology to obtain ${NO}_{2}$ and $O_{3}$ concentration changes between 2020 and 2019 (2020‐2019) for which meteorological impacts have been accounted for is as follows. Previous studies (Fiore et al., 2003; R. F. Silvern et al., 2019; Tai et al., 2012) have shown that GC can reproduce the temporal variability of ${NO}_{2}$, $O_{3}$ and particulate matter, implying that GC accounts for meteorological impacts. We conduct GC simulations for 2020 and 2019 with identical emissions but with the respective meteorology from MERRA‐2 reanalysis. Since, we use the same anthropogenic emission in 2020 and 2019, the GC differences in ${NO}_{2}$ and $O_{3}$ between 2020 and 2019 are solely due to meteorological influences, that is, differences in wind speed, boundary layer height, photo‐chemistry etc.:

(1) $$\Delta NO_{2\left(GC\right)}=NO_{2\left(GC,2020\right)}-NO_{2\left(GC,2019\right)}$$

(2) $$\Delta O_{3\left(GC\right)}=O_{3\left(GC,2020\right)}-O_{3\left(GC,2019\right)}$$

The difference between the 2020 and 2019 ${NO}_{2}$ and $O_{3}$ observations for specific time periods include influence from both meteorological and emissions changes:

(3) $$\Delta NO_{2\left(obs\right)}=NO_{2\left(obs,2020\right)}-NO_{2\left(obs,2019\right)}$$

(4) $$\Delta O_{3\left(obs\right)}=O_{3\left(obs,2020\right)}-O_{3\left(obs,2019\right)}$$

In order to account for the differences resulting from meteorology and isolate the impact resulting from emission changes we subtract the difference in the simulations from the difference in the observations as follow (Qu et al., 2021),

(5) $$\Delta NO_{2\left(acc\right)}=\Delta NO_{2\left(obs\right)}-\Delta NO_{2\left(GC\right)}$$

and similarly for ozone:

(6) $$\Delta O_{3\left(acc\right)}=\Delta O_{3\left(obs\right)}-\Delta O_{3\left(GC\right)}$$

Where, “acc” refers to meteorology accounted for, “obs” refers to in‐situ or TROPOMI measured concentrations, and “GC” refers to GEOS‐Chem model simulated concentrations. This approach results in values that have accounted for meteorological influence to estimate the concentration changes resulting only from COVID‐19 emission reductions.

## 3 Tropospheric NO_2_ and O_3_: Impact of Meteorological Conditions

**Figure 1:**
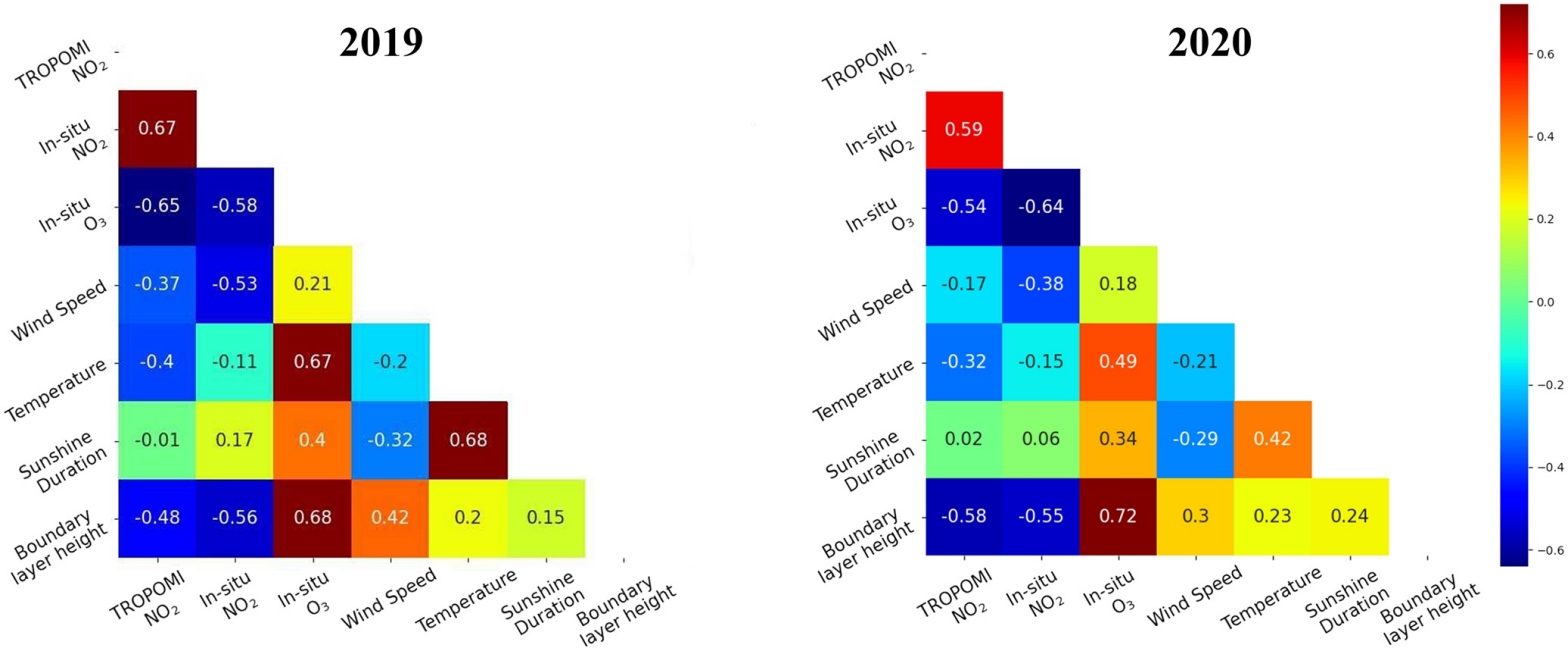
Correlation matrix (R‐correlation coefficient) between meteorological parameters and ${NO}_{2}$ and $O_{3}$ concentrations (January to June in 2020 and 2019) in Munich.

Like previous studies (Çelik & İbrahim, 2007; Deroubaix et al., 2021; Ordóñez et al., 2020; Voiculescu et al., 2020), we investigated correlations between satellite and in‐situ ${NO}_{2}$ and $O_{3}$ concentrations and local meteorological parameters to find the dependency of ${NO}_{2}$ and $O_{3}$ concentrations on meteorology. The correlation matrix is shown in Figure 1 for the Munich metropolitan area. We find similar correlation behavior between variables for 2019 (no lockdown) and 2020 (lockdown). Generally, satellite and in‐situ ${NO}_{2}$ concentrations have a negative correlation with wind speed, temperature and boundary layer height, for example, as pollutants disperse more at high wind speeds than at low wind speeds. As temperature and sunlight increases, the rate of ${NO}_{2}$ photochemical loss accelerates, and the planetary boundary layer expands resulting in higher dilution. $O_{3}$ concentrations have a generally negative correlation with ${NO}_{2}$ concentrations and positive correlation with sunshine duration and temperature. This results from the fact that ${NO}_{2}$ and high solar radiation play an important role in regulating $O_{3}$. Temperature has been shown to have a significant influence on ozone production over Europe under various ${NO}_{X}$ conditions (Coates et al., 2016; Melkonyan & Wagner, 2013). In addition, Curci et al. (2009) show that increasing temperature causes an increase in biogenic VOC emissions, which can raise the ozone level, especially in the summer. Future climate conditions in Europe (as a result of global warming) will almost certainly have an impact on ozone pollution (Engardt et al., 2009; Forkel & Knoche, 2006; Meleux et al., 2007; Vautard et al., 2007). Europe may experience more intense and frequent heatwaves and droughts in the future, which will increase wildfire events and, as a result, background ozone levels will increase (De Sario et al., 2013; Meehl & Tebaldi, 2004). Furthermore, temperature, boundary layer height and solar radiation, which are considered to be the most related meteorological factors influencing ${NO}_{2}$ and $O_{3}$ concentrations, are interdependent.

## 4 Changes in NO_2_ and O_3_ Concentrations in Germany Due To COVID‐19 Lockdown Restrictions

**Figure 2:**
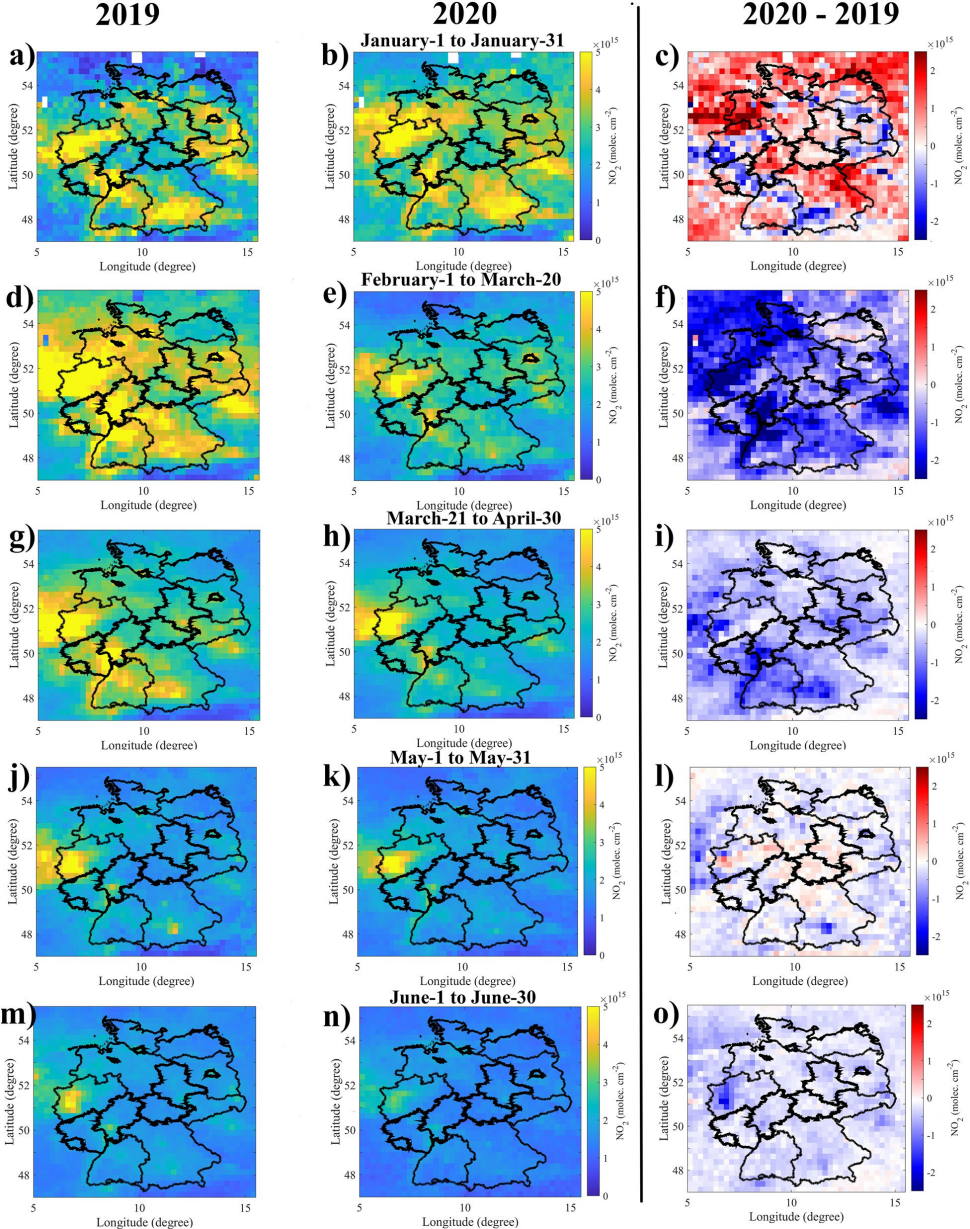
Mean TROPOspheric Monitoring Instrument (TROPOMI) tropospheric ${NO}_{2}$ column densities for 2019 (first column) and 2020 (second column). The absolute differences in TROPOMI tropospheric ${NO}_{2}$ column densities between 2020 and 2019 (third column).

**Figure 3:**
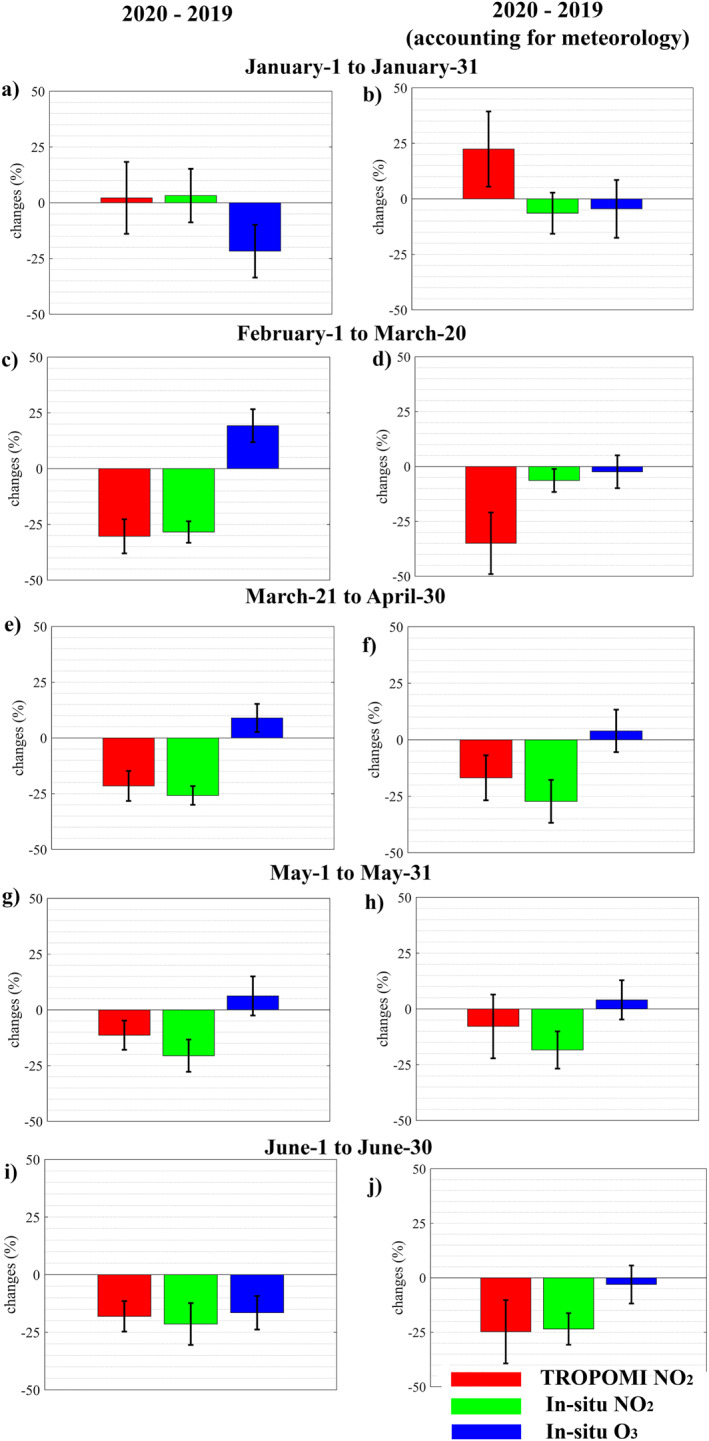
Mean relative changes in meteorological impacts unaccounted (left column) and accounted (right column) ${NO}_{2}$ and $O_{3}$ concentrations in eight metropolitan cities between 2020 and 2019. Error bars represent the 1 σ (standard deviation) of mean of eight metropolitan cities.

In this study, we compare January through June of 2020 and 2019. This time period is divided into five sections: (a) No lockdown restrictions from January 1 to January 31, 2020. (b) No lockdown restrictions with anomalous weather conditions from February 1 to March 20, 2020. (c) Strict lockdown restrictions from March 21 to April 30, 2020 (spring). (d) Loose measures from May 1 to May 31, 2020 (late spring). (e) Loose measures from June 1 to June 30, 2020 (early summer). The mean TROPOMI and in‐situ ${NO}_{2}$ in January of 2020 was slightly higher than in 2019 (Figures 2c and 3a). However, between February 1 and March 20, 2020, prior to the lockdown, mean observed TROPOMI and in‐situ ${NO}_{2}$ was already significantly lower than in 2019 at both the national (Figure 2f) and urban scales (Figures 3c and S5). This can be attributed to unusually high wind speeds caused by storms in February 2020 (DLR COVID‐19, 2020). The first governmental COVID‐19 lockdown restrictions went into effect on March 21, 2020. In the period following the lockdown implementation, lower ${NO}_{2}$ values are observed compared to 2019. In‐situ measurements show lower mean $O_{3}$ concentrations in January and June 2020, and higher mean $O_{3}$ concentrations from February 1 to May 31, 2020, compared to 2019 (Figures 3 and S5).

GC model simulations are used to estimate the difference in ${NO}_{2}$ and $O_{3}$ concentrations between 2020 and 2019 caused by meteorology. Studies (Fiore et al., 2003; R. F. Silvern et al., 2019; Tai et al., 2012) have demonstrated that GC can reproduce the observed temporal variability of ${NO}_{2}$, $O_{3}$ and particulate matter, implying that GC accounts for impacts of meteorology when using precise meteorological data and emission inventories. In our study, we also compare the GC and observed concentrations from 2019 to verify that the GC can reproduce the temporal variability of observed concentration changes. The 2019 (January to June) period is used to validate the GC model simulations as unlike 2020 emissions are not affected by changes resulting from COVID measures. To validate the GC model, we compared GC surface concentrations with in‐situ surface concentrations, and GC column densities with TROPOMI column densities (Figure S6, for cologne metropolitan area). We find good agreement between GC surface ${NO}_{2}$ concentrations and in‐situ surface ${NO}_{2}$ concentrations for eight metropolitan areas (R, pearson correlation coefficient, >0.65, with high R (0.75) for Cologne). Similar results were obtained for GC surface $O_{3}$ concentrations, (R > 0.65, with a high R (0.74) for Dresden). GC underestimates ${NO}_{2}$ surface concentrations, except for Hamburg. The mean bias (GC‐in‐situ) ranges from +2.9% to −23%. Except for Hamburg and Hanover, GC overestimates surface $O_{3}$ concentrations, with mean bias ranges from +24% to −10.3%. When comparing 2019 GC and TROPOMI ${NO}_{2}$ column densities, relatively low correlation (R, between 0.24 and 0.55) was found, and the ${NO}_{2}$ column densities in metropolitan areas were underestimated by GC (mean bias ranges from −4% to −28%). However, the GC model is capable of modeling the spatial variability of ${NO}_{2}$ column densities at the national scale, emphasizing GC's ability to represent the distribution of emission source locations (Figure S7). The over/under estimation of ${NO}_{2}$ and $O_{3}$ concentrations are caused by the emission inventory (over/under estimation of emission) used in GC simulation. The low bias in ${NO}_{2}$ and high bias in $O_{3}$ could be consistent with ${NO}_{X}$ saturated conditions. Because we use the difference in GC concentrations between 2020 and 2019 ($\Delta \;{NO}_{2\left(GC\right)}$ and $\Delta \;O_{3\left(GC\right)}$), general biases are canceled out.

Due to the passage of two strong storm systems February 2020 experienced high winds. We consider the period from February 1 to March 20, 2020 (prior to the implementation of lockdown restrictions) to determine the extent to which meteorology is responsible for variations in pollutant concentrations. Before accounting for meteorology, the difference in mean in‐situ ${NO}_{2}$ concentration between 2020 and 2019 is −28% for the period February 1 and March 20. After accounting for meteorology, the difference is reduced to −6% (consistent with meteorology accounted changes for the period between January 1 and January 31, 2020 compared to 2019, Figures 1a–1d). This emphasizes the significance of employing our method to account for meteorological impacts. The impacts of meteorology on in‐situ and TROPOMI ${NO}_{2}$ concentrations are relatively small (+0.4% and −0.6%, respectively) for the period between March 21 and June 30, 2020 (the period after the implementation of lockdown restrictions). After accounting for meteorology, the mean in‐situ and TROPOMI ${NO}_{2}$ values between March 21 and June 30, 2020 were significantly lower (by 23% and 16%, respectively) than the same period in 2019 (Figures 3f, 3h, and 3j)). Other studies (Barré et al., 2020; Grange et al., 2020; Solberg et al., 2021) that used a machine learning and statistical approach to account for meteorological impacts also found that the impact of the COVID‐19 pandemic on ${NO}_{X}$ emissions lasted at least until June 2020. After accounting for meteorology, we observed a slight increase in mean in‐situ $O_{3}$ concentration between March 21 and May 31, 2020 (consistent with Deroubaix et al., 2021; Ordóñez et al., 2020), and a slight decrease in mean in‐situ $O_{3}$ concentration in June 2020 compared to 2019. In our study areas (metropolitan areas), the impact of meteorological conditions on in‐situ $O_{3}$ concentrations are clearly noticeable in all periods. Meteorological conditions were favorable for high $O_{3}$ concentrations between February 1 and May 31, 2020 (consistent with Gaubert et al., 2021), while meteorological conditions were favorable for low $O_{3}$ concentrations in January and June 2020. For instance, before accounting for meteorology, mean $O_{3}$ concentration in June 2020 is 16.5% lower than in 2019, which could be attributed to the low temperature (less ozone production) in June 2020 (Figure S8j). After accounting for meteorology, the difference between mean $O_{3}$ concentrations in June 2020 and the same period in 2019 is reduced to −3%. Meteorology had a different impact on ${NO}_{2}$ and $O_{3}$ levels and this impact also varied for different time periods. This demonstrates the complex relationship between $O_{3}$, ${NO}_{2}$, and meteorological conditions.

We found large discrepancies between in‐situ and TROPOMI ${NO}_{2}$ changes for the study period. It is important to note that the number of TROPOMI cloud‐free measurements between 2020 and 2019 may have an impact on results (for Munich, TROPOMI measurements are available for 269 days out of 363 days). In addition, the TROPOMI overpass occurs at 13.30 local time, which may make it less sensitive to traffic‐related emissions (peak in the morning from 7 to 9 a.m and evening from 4 to 7 p.m). We conducted two comparisons between 2019 in‐situ ${NO}_{2}$ and TROPOMI ${NO}_{2}$ measurements to determine whether the TROPOMI measurements (overpass occurs around 13.30) could represent traffic‐related emissions. First, we compare the mean 24 h in‐situ ${NO}_{2}$ to the TROPOMI ${NO}_{2}$ observation. Second, we compare the in‐situ ${NO}_{2}$ at the time of TROPOMI overpass with the TROPOMI ${NO}_{2}$, which should have better agreement. We use the empirical relationship (Lorente et al., 2019) that includes boundary layer information to convert the surface concentration to column density. The TROPOMI observations agree well with the in‐situ observations at the TROPOMI overpass time (mean bias (TROPOMI ‐ in‐situ) is about −13%), whereas TROPOMI underestimates ${NO}_{2}$ compared to the 24‐h mean in‐situ value (mean bias is about −41.5%) (Figure S9, for Munich). This indicates that TROPOMI is not suitable to directly represent the 24‐h mean (daily concentration), which could lead to errors in estimating lockdown effects, because the lockdown primarily reduced traffic‐related emissions. Furthermore, the observed satellite column concentration is certainly influenced by the background concentration. The free tropospheric background contributes 70%–80% of the total column observed via satellite (R. Silvern et al., 2018; Travis et al., 2016). R. F. Silvern et al. (2019) and Qu et al. (2021) demonstrate the importance of accounting for the influence of free tropospheric ${NO}_{2}$ background on satellite column measurements to infer the changes in surface ${NO}_{X}$ emission. The primary sources of background ${NO}_{2}$ are lightning, soil, wildfires and long‐range transport of pollution (L. Zhang et al., 2012), which are unaffected by lockdown restrictions. The contribution from soil has been shown to increase up to 27% of total ${NO}_{X}$ emissions at elevated temperatures (Butterbach‐Bahl et al., 2001) (discussed below). In addition, subtracting the contribution of the ${NO}_{2}$ background from satellite column observation is complex, because of its non‐uniformity (Marais et al., 2018, 2021), thus, using column measurements is challenging for estimates of local changes in ${NO}_{2}$ emissions. In contrast to satellite column measurements, background ${NO}_{2}$ has little influence (5%–10%) on in‐situ surface ${NO}_{2}$ concentrations (R. F. Silvern et al., 2019). The discrepancies between in‐situ and TROPOMI changes primarily results from unaccounted background ${NO}_{2}$ influence on the satellite observation and that TROPOMI's overpass time makes it less sensitive to overall diurnal emissions. These discrepancies limit the use of satellite column measurements to infer the surface ${NO}_{X}$ emission changes.

**Figure 4:**
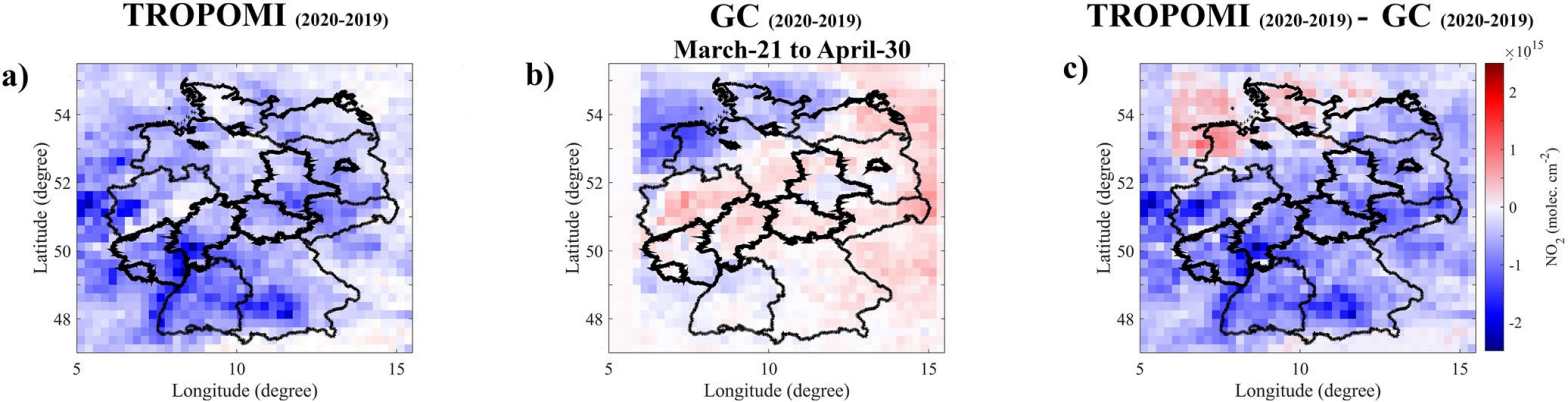
(a) The absolute difference in TROPOspheric Monitoring Instrument and (b) GEOS‐Chem ${NO}_{2}$ column densities between 2020 and 2019 (stricter lockdown period: March 21–April 30). The absolute difference between first two columns is shown in panel (c).

The ${NO}_{2}$ column densities in rural locations were also investigated. During the 2020 stricter lockdown period, after accounting for meteorology, slightly increased ${NO}_{2}$ vertical column densities over North‐West Germany are observed compared to 2019 (Figure 4c). We hypothesize that this is due to enhanced soil ${NO}_{X}$ emissions over North‐West Germany in the 2020 stricter lockdown period (associated with increased temperature over North‐West Germany (Figure S8f); soil ${NO}_{X}$ emissions typically increase with temperature (Oikawa et al., 2015). Soil ${NO}_{X}$ emissions are expected to be high in the early spring (stricter lockdown period), even though the average temperature in May and June is higher than in the stricter lockdown period, because agricultural practices such as fertilizer application begin and end in the early spring (Ramanantenasoa et al., 2018; Viatte et al., 2020). Fertilized soils have high potential for ${NO}_{X}$ emissions (Almaraz et al., 2018; S. Liu et al., 2017; Skiba et al., 2021). Figure S11 shows the monthly mean ${NH}_{3}$ total column densities over Germany. High ${NH}_{3}$ total column densities were observed in April as agricultural practices (fertilizer application) began in the early spring. Notably, strong enhancements were observed over North‐West Germany. The total column of ${NH}_{3}$ over North‐West Germany in 2020 (strict lockdown period) is higher than in 2019 (Figure S12). This supports our hypothesis that North‐West Germany, which is dominated by Grass and Crop land (ESA CCI, 2020), is an agricultural region, with fertilized soil producing ${NO}_{X}$ in elevated‐temperature environments.

## 5 Ozone Sensitivity to NO_X_ Changes

**Figure 5:**
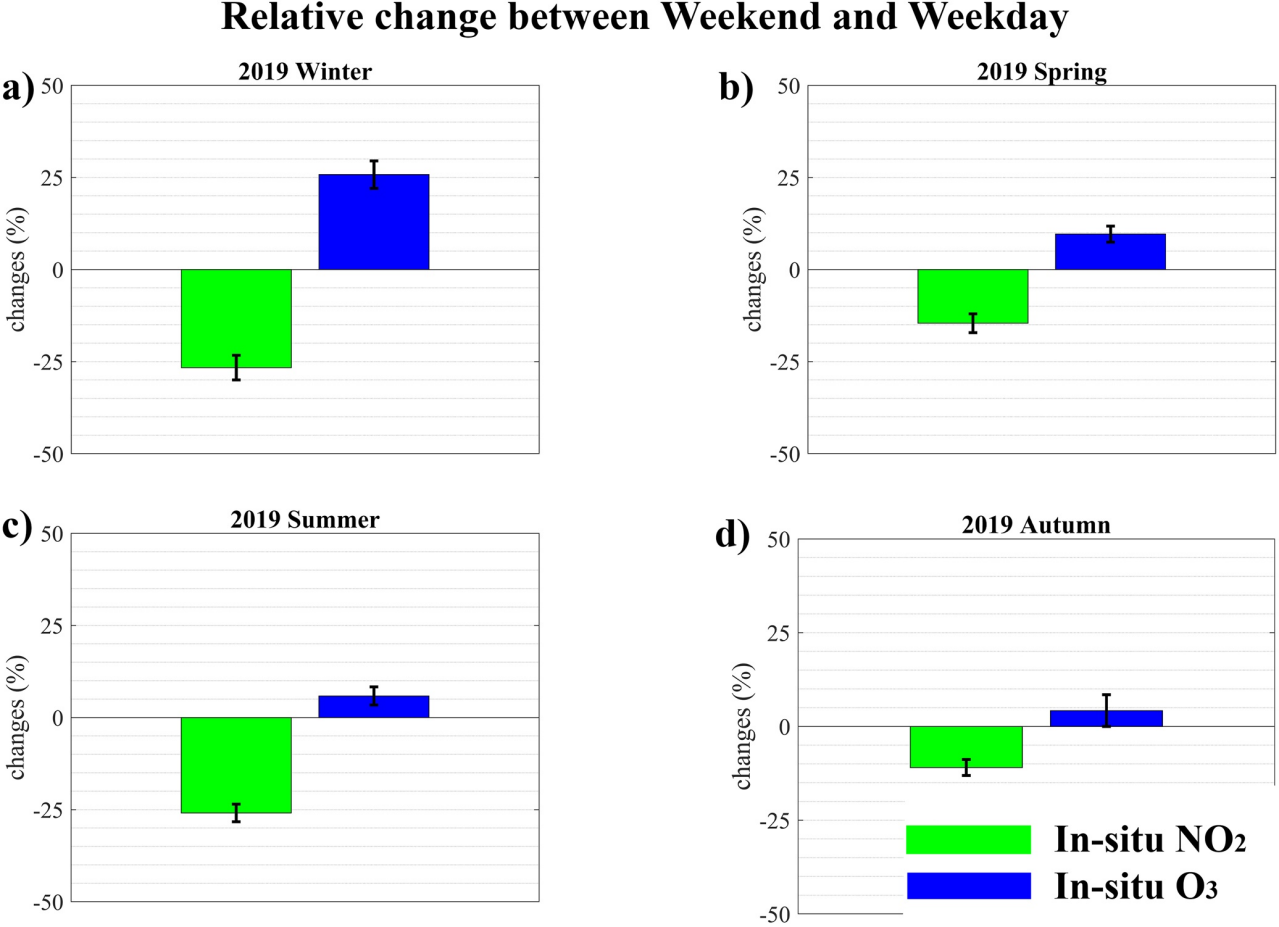
Mean relative change in in‐situ ${NO}_{2}$ and $O_{3}$ concentrations in Munich between weekends and weekdays. Error bars represent statistical uncertainty (1 σ) in the calculation of relative change between weekend and weekday means.

Like previous studies that reported the urban ${NO}_{2}$ weekly cycle (Beirle et al., 2003; Ialongo et al., 2020), we also investigate this at the national (Germany) and urban scale (Figures S13 and S14). Both TROPOMI and in‐situ ${NO}_{2}$ measurements show that weekend ${NO}_{2}$ concentrations are lower than weekday ${NO}_{2}$ concentrations, because primary emission activities such as transportation typically decrease on weekends. Studies (Sicard et al., 2020; Wang et al., 2014) have demonstrated that analyzing the difference in weekday versus weekend $O_{3}$ concentrations helps identify the ozone production regime. As ${NO}_{X}$ emissions decrease on weekends, the response of ozone will demonstrate whether ozone production is ${NO}_{X}$ limited or saturated. Hammer et al. (2002); Gaubert et al. (2021) used the $H_{2}O_{2}$/${HNO}_{3}$ ratio and Sillman et al. (2003) used the $O_{3}$/${NO}_{y}$ ratio as a way to identify the ozone production regime over Europe urban regions. Previous studies (Beekmann & Vautard, 2010; Derwent et al., 2003; Gabusi & Volta, 2005; Gaubert et al., 2021; Martin et al., 2004) have demonstrated that European urban regions are characterized as ${NO}_{X}$ saturated ozone production regime. The influence of biogenic VOC emissions on ozone is relatively low in Europe (Curci et al., 2009; Simpson, 1995). There also is a shift between ${NO}_{X}$ saturated and ${NO}_{X}$ limited regimes during the year; in the winter, ozone production is usually ${NO}_{X}$ saturated, whereas it is often ${NO}_{X}$ limited in the summer (Jin et al., 2017). The winter and spring $O_{3}$ weekend effect is much stronger than the summer $O_{3}$ weekend effect (Figure 5, for Munich metropolitan area); reduced ${NO}_{X}$ emission on weekends increase $O_{3}$ concentrations, that is, ${NO}_{X}$ saturated conditions prevail, consistent with above mentioned previous studies, which shows that ${NO}_{X}$ saturated conditions persist to the current time period. Therefore, German metropolitan areas are expected to be in a ${NO}_{X}$ saturated ozone production regime also during the initial 2020 COVID‐19 pandemic period. Notably, we found an increase (4%) in meteorology accounted for mean in‐situ $O_{3}$ concentrations in spring (March 21 and May 31, 2020) and a slight decrease (3%) in meteorology accounted for mean in‐situ $O_{3}$ concentrations in early summer (June, 2020) compared to the same period in 2019. This implies that the degree of ${NO}_{X}$ saturation of ozone production is weakening from winter to summer (consistent with weekend effects and Jin et al., 2017; Kang et al., 2021). During the lockdown period, the daily maximum 8‐h mean $O_{3}$ concentration in metropolitan areas also exceeded the EU target value (120 μg/$m^{3}$) (2 days in Munich, Berlin, Cologne, Stuttgart metropolitan areas). These exceedances are more attributable to ${NO}_{X}$ saturated conditions than to meteorology.

## 6 Conclusions

A year‐to‐year comparison of atmospheric pollutant concentrations is widely used to estimate the influence of reductions in anthropogenic emissions on atmospheric pollutant concentration changes during the COVID‐19 pandemic period. However, these findings could be misleading if meteorological impacts are not taken into account. We used identical anthropogenic emissions in 2020 and 2019 in GC model simulations, allowing us to separate the changes in ${NO}_{2}$ and $O_{3}$ attributed to meteorological impacts from the observed changes. Finally, we show that, due to reductions in anthropogenic emissions during the COVID‐19 pandemic period, meteorology accounted for mean in‐situ & TROPOMI ${NO}_{2}$ concentrations decreased by 23% & 16%, respectively, compared to 2019, in eight German metropolitan cities between March 21 and June 30. After accounting for meteorology, we find a nationwide decrease in TROPOMI ${NO}_{2}$ concentrations except for North‐West Germany, which can be attributed to enhanced ${NO}_{X}$ emissions from agricultural soils during the 2020 stricter lockdown period. We hypothesize that North‐West Germany is a hot spot of soil ${NO}_{X}$ emissions in elevated‐temperature environments due to intensive agricultural practices (fertilizer applications) during the early spring. The IASI ${NH}_{3}$ satellite data also supports our statement that North‐West Germany is an intensive agricultural region during the early spring.

After accounting for meteorology, the concentration of $O_{3}$ increased slightly (4%) during the 2020 spring lockdown while it decreased slightly (3%) during the 2020 early summer lockdown, in response to decreased ${NO}_{2}$ in both time periods, compared to 2019. This implies that the degree of ${NO}_{X}$ saturation of ozone production is weakening from winter to summer. These findings are also supported by the response of $O_{3}$ to changes in precursor emissions using weekend versus weekday differences. Therefore, reducing ${NO}_{X}$ emissions would benefit summer ozone reduction, whereas reducing ${NO}_{X}$ emissions would increase ozone levels during winter and spring. Appropriate ${NO}_{X}$ and VOCs emission control strategies are required to mitigate ozone pollution in German metropolitan areas during winter and spring; otherwise, it may lead to incorrect environmental regulation policies that are closely linked to public health. Despite a sharp decrease in emissions from the transportation sector, emissions from natural sources (dust storms, wildfires) and agriculture sectors were unaffected by 2020 COVID‐19 lockdown restrictions. Changes in other pollutants such as ${PM}_{10}$, ${SO}_{2}$, CO and anthropogenic VOCs (primary pollutant) and ${PM}_{2.5}$ (secondary pollutant) may provide further insight on air quality during the COVID‐19 pandemic period. Extensive studies on air quality during the lockdown period could pave the way for an improved understanding of pollution formation. Those findings will be useful in understanding how reductions in primary emissions affect secondary pollutant formation.

## Conflict of Interest

The authors declare no conflicts of interest relevant to this study.

## Supporting information

Supporting Information S1

## Data Availability

The TROPOMI ${NO}_{2}$ data can be found at https://s5phub.copernicus.eu/. The IASI ${NH}_{3}$ data are available at https://iasi.aeris-data.fr/catalog/. Hourly ${NO}_{2}$ and $O_{3}$ concentrations are downloaded from UBA's website (https://www.umweltbundesamt.de/en/data). Hourly ERA5 meteorological data are freely available at https://cds.climate.copernicus.eu/.
