# Supplementary material for: Tropospheric NO2 and O3 Response to COVID‐19 Lockdown Restrictions at the National and Urban Scales in Germany
Source: J Geophys Res Atmos. 2021 Sep 27;126(19):e2021JD035440. doi: 10.1029/2021JD035440 (PMC8667658; doi:10.1029/2021JD035440)
Supplement: Supplementary file 1 — Supporting Information S1 [file JGRD-126-e2021JD035440-s001.pdf]

Supporting Information for "Tropospheric NO_2 and O_3 response to COVID-19 lockdown restrictions at the national and urban scales in Germany".

Vigneshkumar Balamurugan[1], Jia Chen[1], Zhen Qu[2], Xiao Bi[1], Johannes Gensheimer[1], Ankit Shekhar[3], Shrutilipi Bhattacharjee[4], Frank N. Keutsch[2,5]

[1]TUM Department of Electrical and Computer Engineering, Technische Universität München, Munich, Germany

[2]School of Engineering and Applied Science, Harvard University, Cambridge, Massachusetts, USA

[3]Department of Environmental Systems Science, ETH Zurich, Switzerland

[4]Department of Information Technology, National Institute of Technology Karnataka, Surathkal, India

[5]Department of Chemistry and Chemical Biology, Harvard University, Cambridge, Massachusetts, USA

Contents of this file

1. Figures S1 to S13

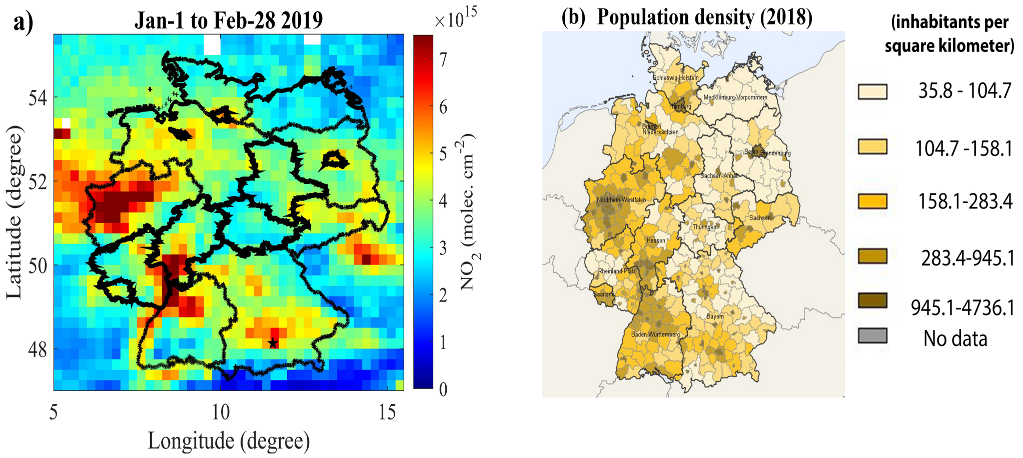

Figure S1. Spatial relationship between the TROPOMI NO_2 concentration (mean NO_2 concentration between January 1 and February 28, 2019) and population density. Population density map was retrieved from Statistisches Bundesamt [© Federal and State Statistical Offices, Germany, 2020].

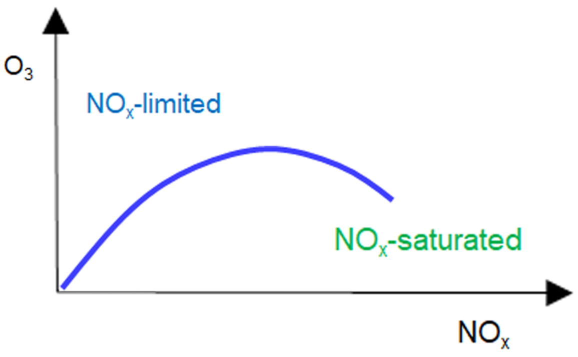

Figure S2. Graphical representation of NO_X saturated and NO_X limited ozone production regime.

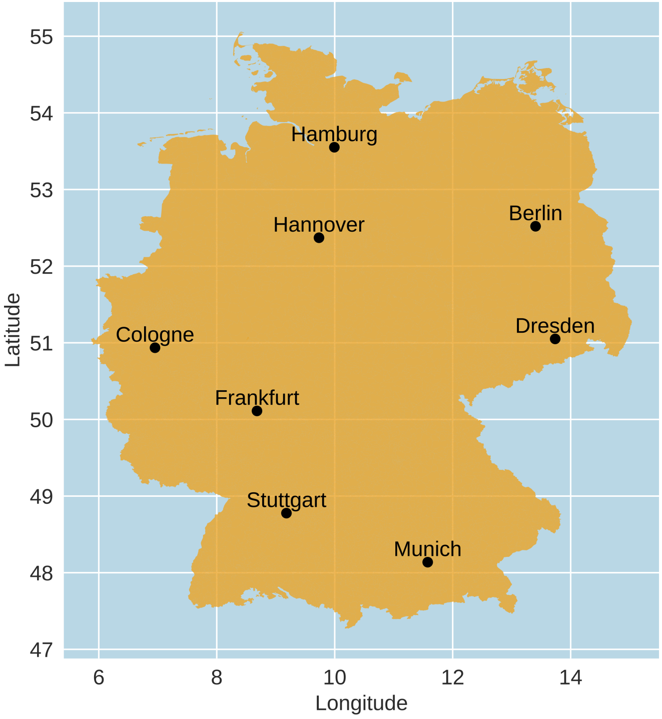

Figure S3. Visualization of the location of the urban study regions.

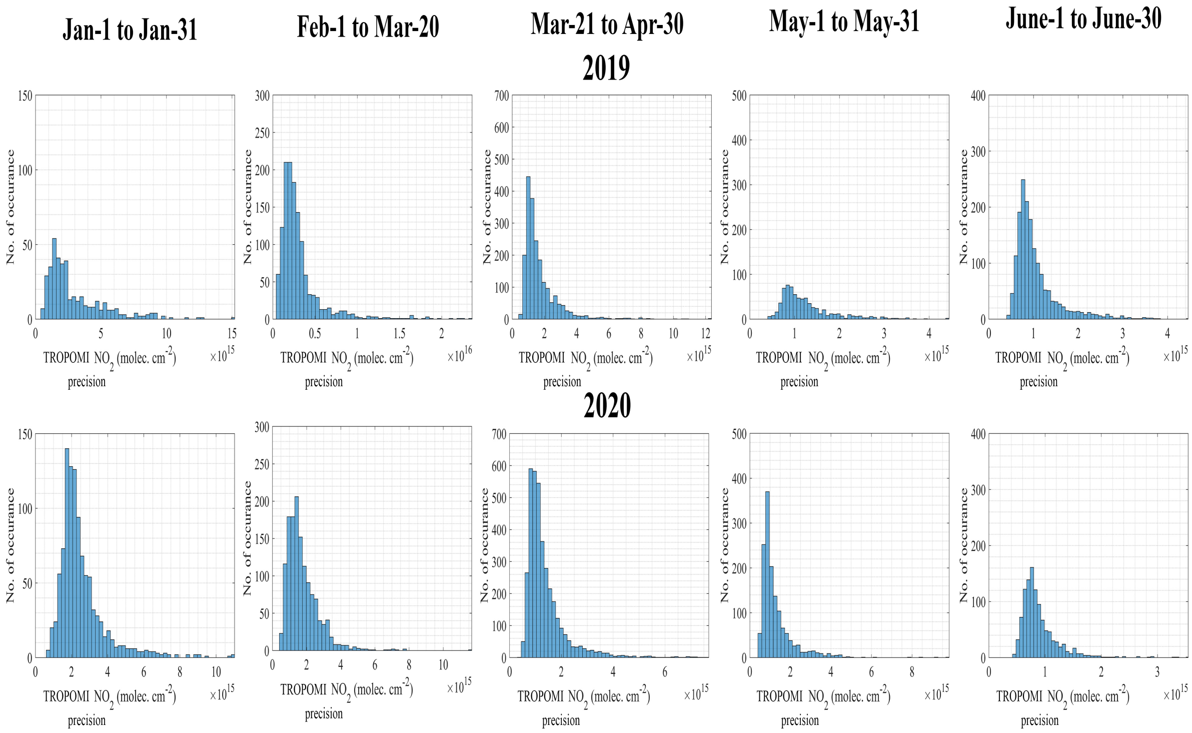

Figure S4. TROPOMI uncertainty distribution for different study period in 2019 (top) and 2020 (bottom).

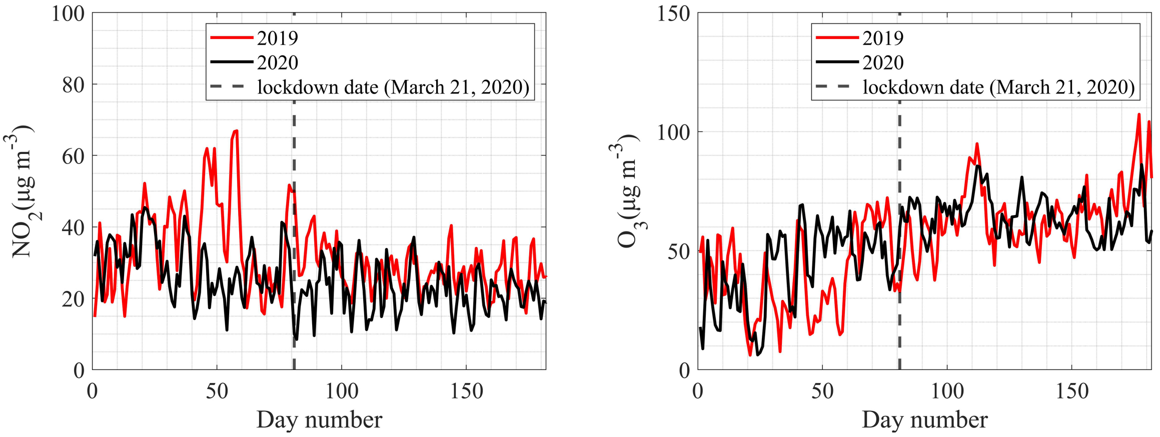

Figure S5. Mean of eight metropolitan area's in-situ NO_2 (left) and O_3 (right) concentrations in 2019 and 2020.

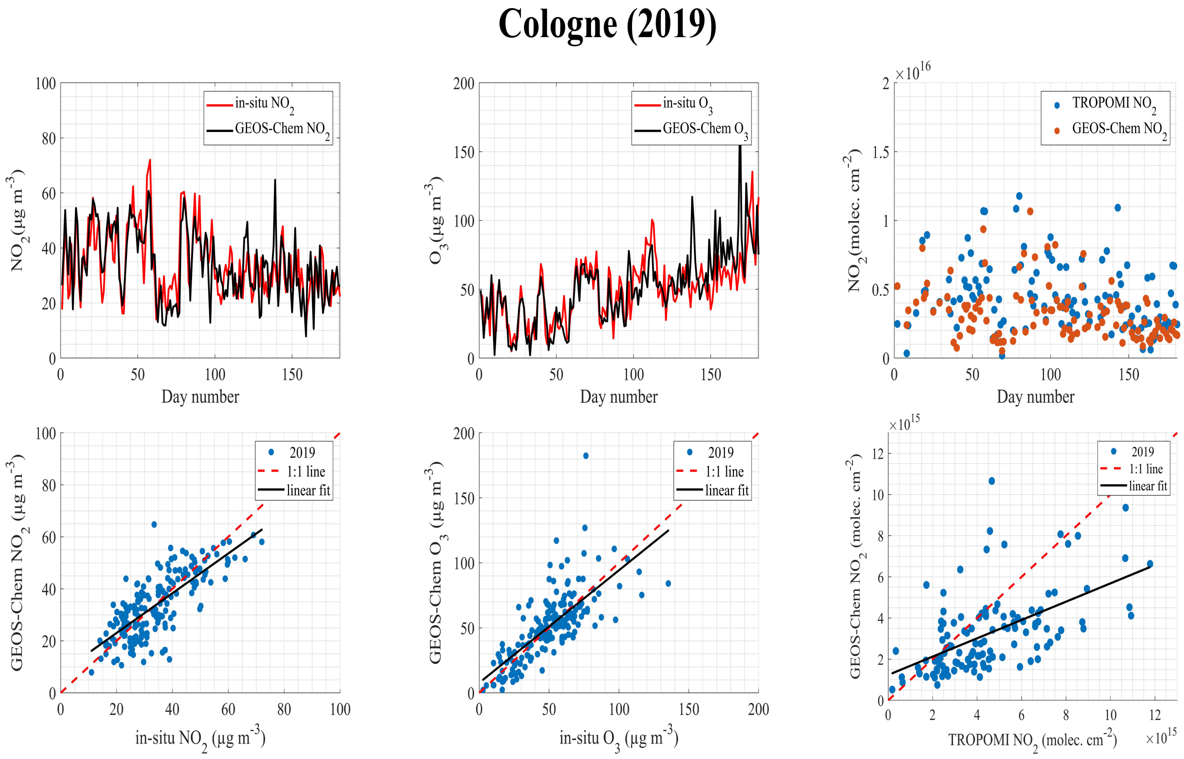

Figure S6. Comparison between 2019 GEOS-Chem vs in-situ NO_2 and O_3 (left and middle), and 2019 GEOS-Chem vs TROPOMI NO_2 (right) for Cologne metropolitan area.

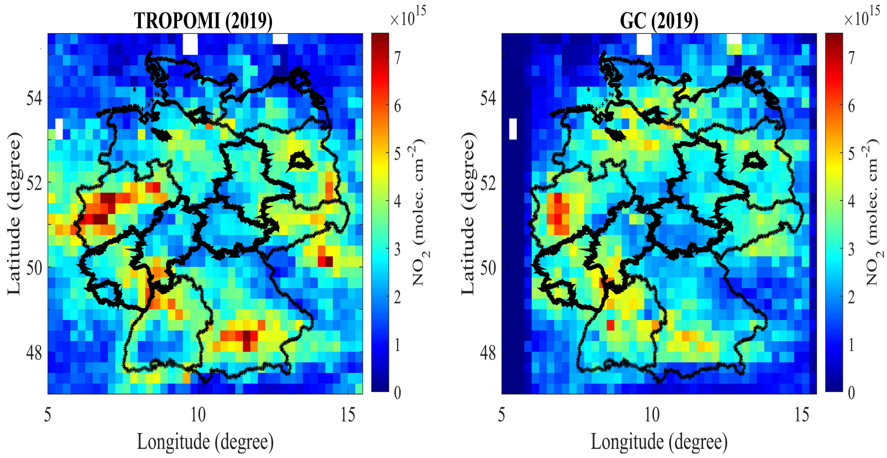

Figure S7. Mean TROPOMI NO_2 and GEOS-Chem NO_2 column densities in 2019 (January to June) at national scale.

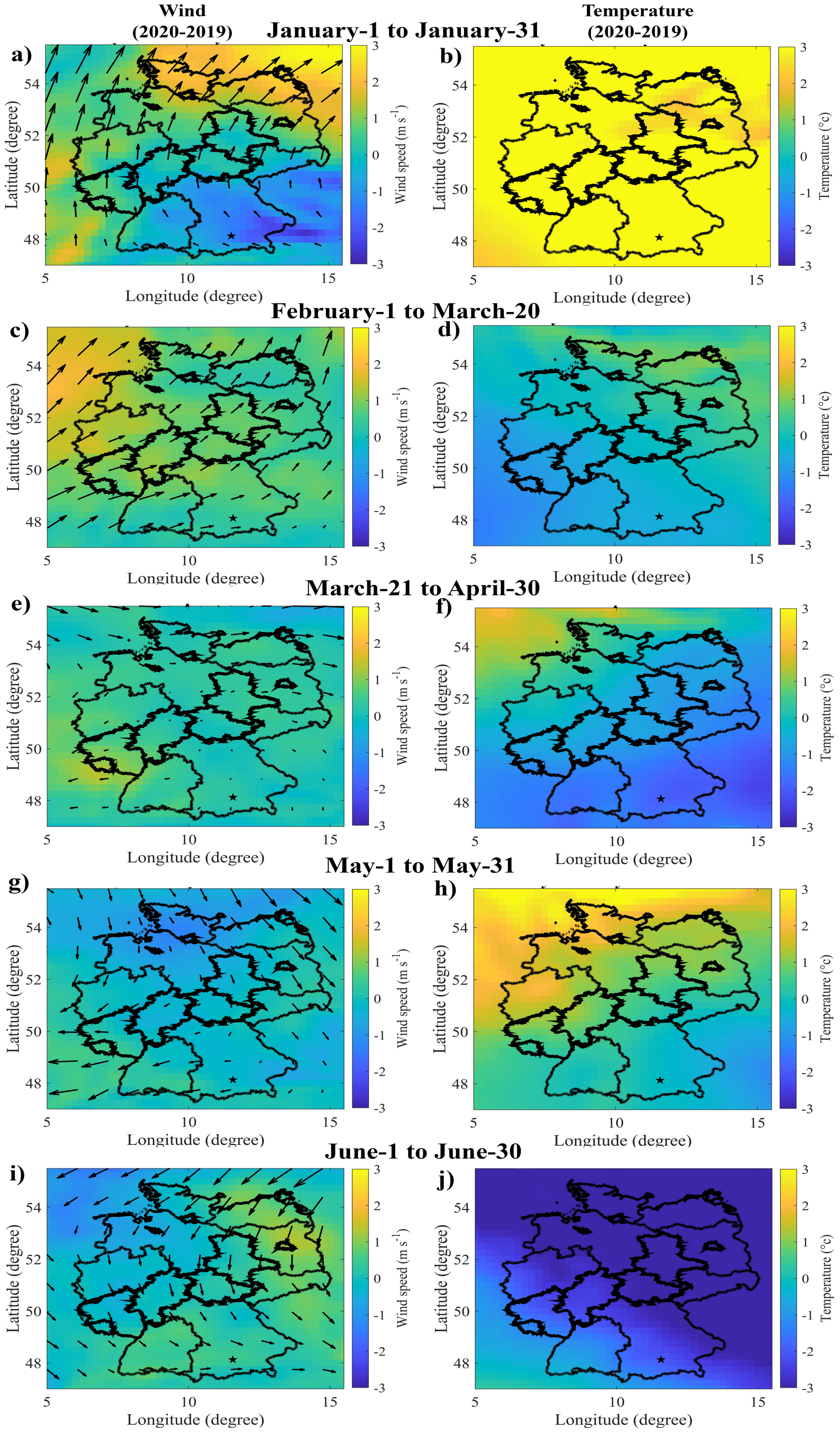

Figure S8. The absolute difference in wind condition (left) and temperature (right) between 2020 and 2019 (2020-2019). Wind speed difference and temperature difference is plotted in 0.25-degree grid, whereas, wind direction difference (black arrow) is plotted in 1-degree grid.

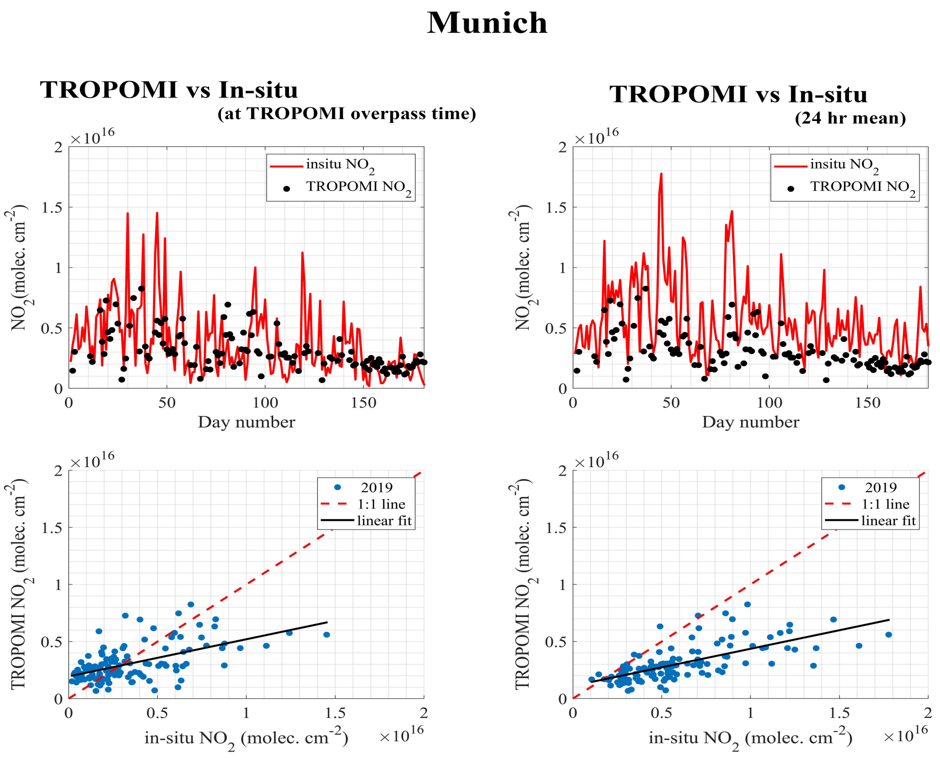

Figure S9. Comparison between TROPOMI NO_2 and in-situ NO_2 (column converted) at TROPOMI overpass time (left). Comparison between TROPOMI NO_2 and 24 hour mean in-situ NO_2 (column converted) (right).

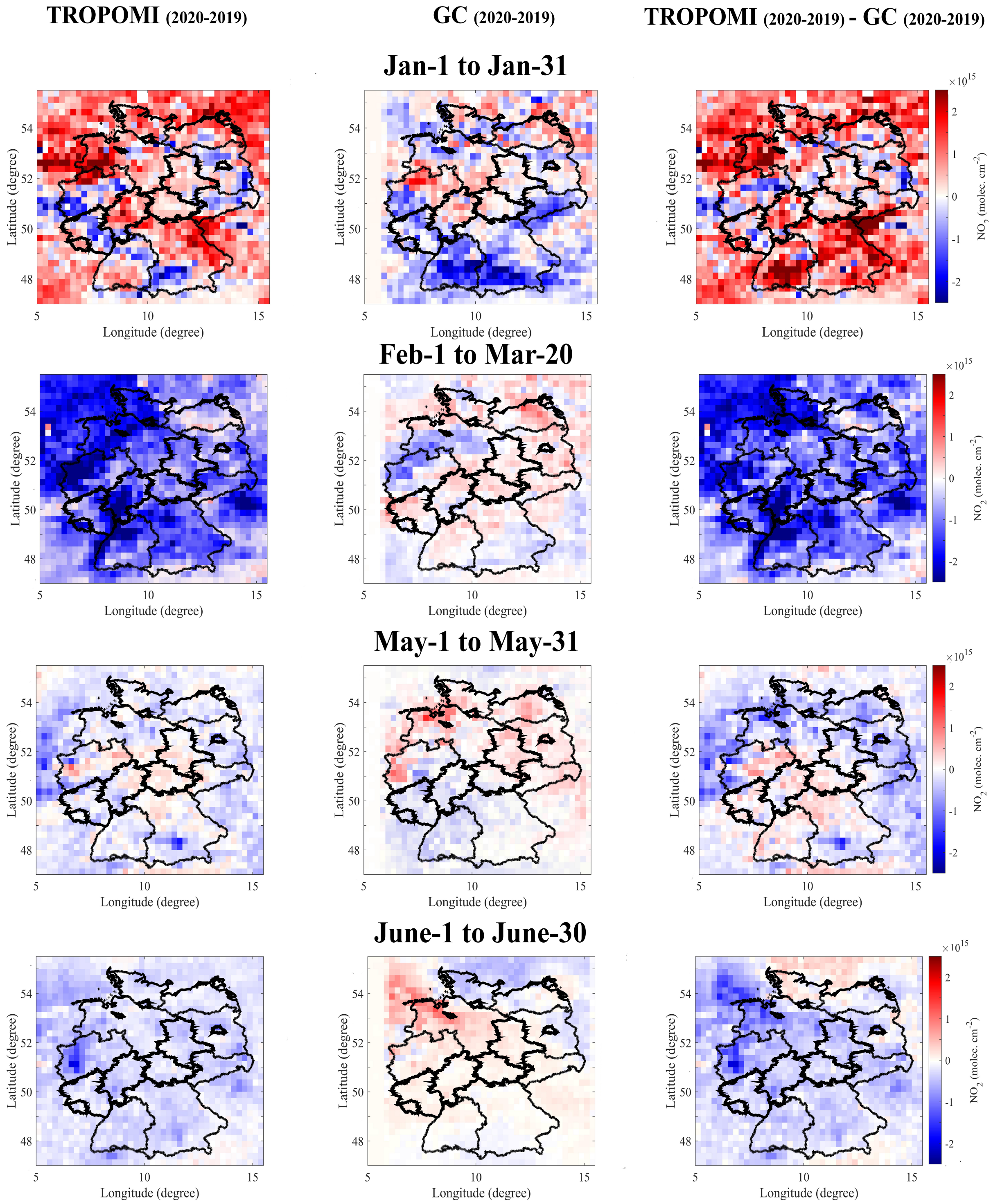

Figure S10. Same as Figure 4, but for different time period.

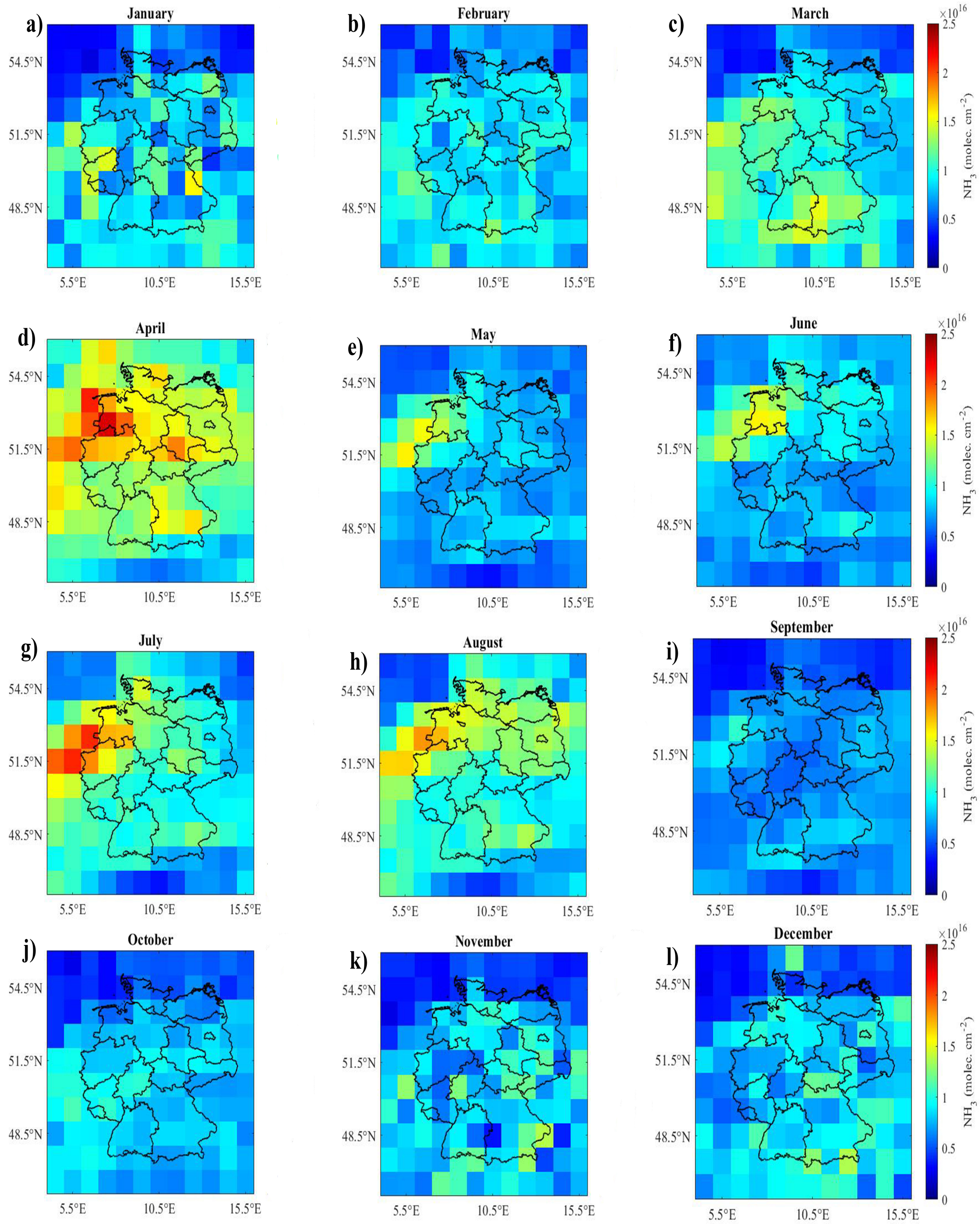

Figure S11. Monthly mean NH_3 total columns measured by IASI for the period from 2018 January to 2020 June.

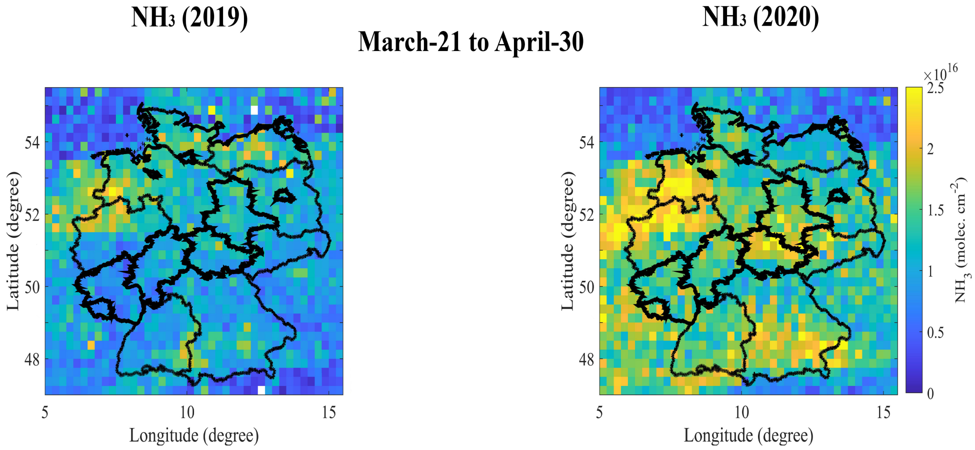

Figure S12. Mean NH_3 total column measured by IASI in 2019 (left) and in 2020 (right). Daily IASI NH_3 measurements gridded at 0.25 degree resolution.

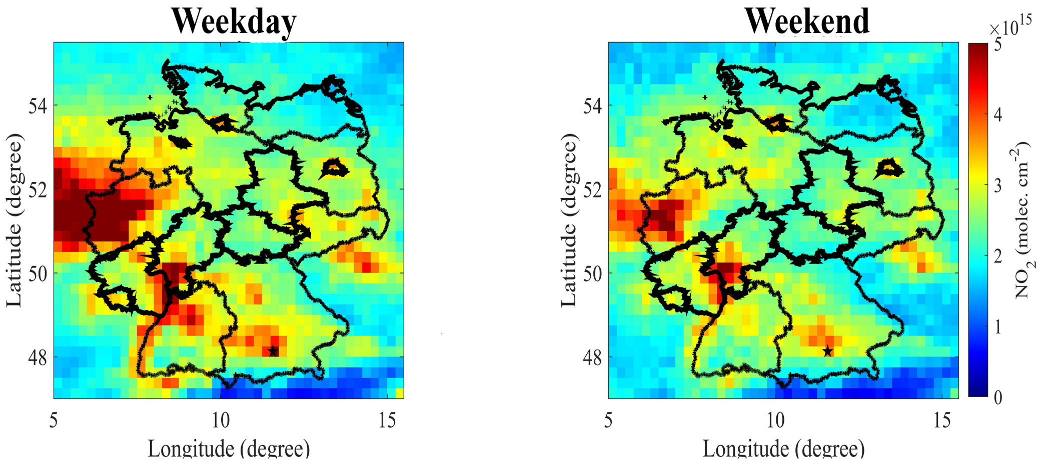

Figure S13. Mean of 2019 (from January to June) TROPOMI NO_2 measurements on weekdays and weekends in Germany.

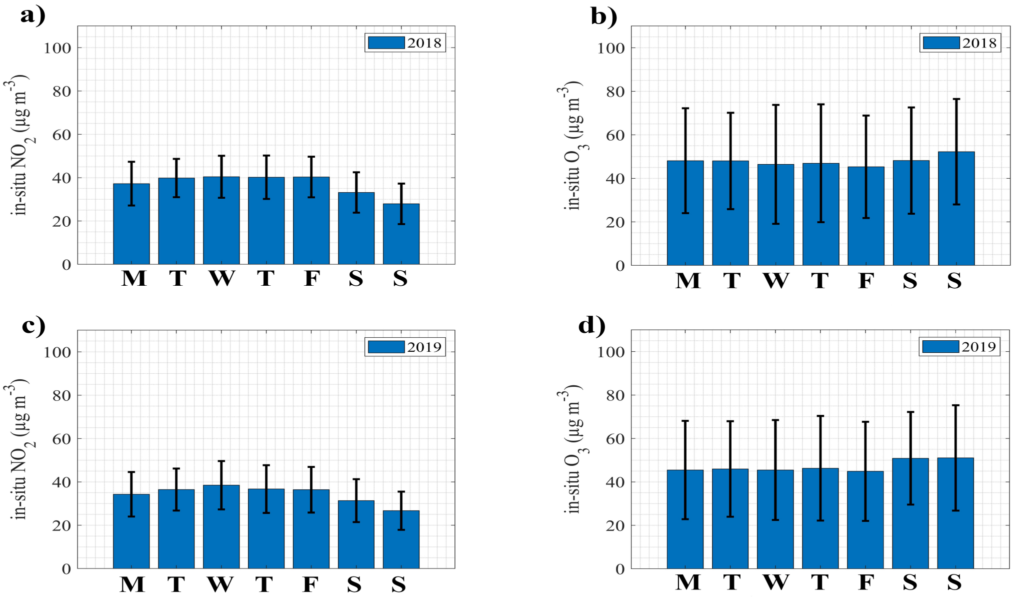

Figure S14. Weekly cycle of 2018 and 2019 in-situ NO_2 and O_3 concentration in Munich. Error bars represent the 1 σ of mean of respective days.
